# Ligandomes obtained from different HLA-class II-molecules are homologous for N- and C-terminal residues outside the peptide-binding cleft

**DOI:** 10.1007/s00251-019-01129-6

**Published:** 2019-09-13

**Authors:** Arieke S.B. Kampstra, Jurgen van Heemst, George M. Janssen, Arnoud H. de Ru, Menno van Lummel, Peter A. van Veelen, René E.M. Toes

**Affiliations:** 1 Department of Rheumatology, Leiden University Medical Center, Leiden, The Netherlands; 2 Center of Proteomics and Metabolomics, Leiden University Medical Center, Leiden, The Netherlands; 3 Department of Immunohematology and Blood Transfusion, Leiden University Medical Center, Leiden, The Netherlands

**Keywords:** Human leucocyte antigen, Peptidome, Cleavage sites, Peptide flanking regions, Protein processing, Antigen presenting cells

## Abstract

Human CD4+ T lymphocytes play an important role in inducing potent immune responses. T cells are activated and stimulated by peptides presented in human leucocyte antigen (HLA)-class II molecules. These HLA-class II molecules typically present peptides of between 12 and 20 amino acids in length. The region that interacts with the HLA molecule, designated as the peptide-binding core, is highly conserved in the residues which anchor the peptide to the molecule. In addition, as these peptides are the product of proteolytic cleavages, certain conserved residues may be expected at the N- and C-termini outside the binding core. To study whether similar conserved residues are present in different cell types, potentially harbouring different proteolytic enzymes, the ligandomes of HLA-DRB1*03:01/HLA-DRB > 1 derived from two different cell types (dendritic cells and EBV-transformed B cells) were identified with mass spectrometry and the binding core and N- and C-terminal residues of a total of 16,568 peptides were analysed using the frequencies of the amino acids in the human proteome. Similar binding motifs were found as well as comparable conservations in the N- and C-terminal residues. Furthermore, the terminal conservations of these ligandomes were compared to the N- and C-terminal conservations of the ligandome acquired from dendritic cells homozygous for HLA-DRB1*04:01. Again, comparable conservations were evident with only minor differences. Taken together, these data show that there are conservations in the terminal residues of peptides, presumably the result of the activity of proteases involved in antigen processing.

**Electronic supplementary material:**

The online version of this article (10.1007/s00251-019-01129-6) contains supplementary material, which is available to authorized users.

## Introduction

Human leucocyte antigen (HLA)-class II molecules are heterodimers consisting of an alpha and beta chain presenting peptide ligands on the surface of professional antigen-presenting cells to CD4^+^ T cells (Brown et al. 1993; Stern et al. 1994).

Activated CD4^+^ T cells play an essential role in the adaptive immune system and mediate multiple functions including the provision of helper activity to B cells to stimulate antibody production. In addition, these T cells can also produce effector molecules including many different cytokines that can shape immune responses. A proper CD4^+^ T cell response is crucial for the immune system to combat infections but plays an important role in anti-tumour responses as well (Jones et al. 2006; Melief and van der Burg 2008; Swain et al. 2012).

CD4^+^ T cells can also play a detrimental role in immunity. In allergy, CD4^+^ T cells targeting allergens are instrumental for class-switching towards an IgE response (Wambre et al. 2012). In autoimmunity, CD4^+^ T cells are important effector cells as they could cause tissue damage, can help the priming of autoreactive CD8^+^ T cell responses and can be involved in the production of autoantibodies (Jones et al. 2006). The contribution to auto-immunity is also reflected by the association with the HLA-class II locus to many different autoimmune diseases (Shiina et al. 2004). Likewise, the HLA-system and CD4^+^ T cells play an important role in allograft rejection (Ali et al. 2013).

Since the early 1990s, many groups have studied the peptide-binding repertoire of HLA-class II molecules (Chicz et al. 1992; Hunt et al. 1992; Rudensky et al. 1991). From these studies, it became clear that this repertoire substantially differs from HLA-class I molecules. For instance, HLA-class II ligands are highly heterogeneous in size and are found in nested sets. The identification of HLA-class II ligands was used to determine the anchor residues of the peptides which interact with the HLA molecule. As these anchor residues differ between different HLA-class II molecules, each individual HLA molecule presents a distinct peptide-binding repertoire (Bondinas et al. 2007). These repertoires and the associated peptide-MHC anchor residues could, as a result, help to predict potential T cell epitopes.

HLA-class II ligands are generated upon antigen-processing by specific proteases (e.g., cathepsins) in the endosomal or lysosomal compartments in contrast to the HLA-class I ligands which are generated by the proteasome (Delamarre et al. 2005). Therefore, we questioned whether the specificity of the proteases would be reflected in amino acid (aa) conservations on the N- and C-terminal side of the presented ligandome.

A few studies have addressed the N-terminal and C-terminal cleavage sites of peptides accommodated in different HLA-class II molecules (mainly HLA-DR) expressed on dendritic cells (DC) (both monocyte-derived and cell line-derived DCs) using a relatively limited number of peptides (Ciudad et al. 2017; Mommen et al. 2016). In these studies, it has been shown that proline is highly frequent at position NP2′ and CP2 when proteins are processed in the endo-lysosomal pathway of mature monocyte-derived DCs, whereas aspartic acid at NP1 and lysine at CP1 are abundantly present when processed in the cytosol (Ciudad et al. 2017). In these studies it was indicated that these observations corresponded to the substrate preferences of cathepsins and metalloproteases but also caspases and granzymes, based on the MEROPS database. In contrast, another study showed higher frequency of aliphatic (especially Leucine) and acidic amino acids at NP1 and CP1 of eluted peptides from MUTZ-3-derived DCs, though lacking a proline at NP2′ and CP2 (Mommen et al. 2016). These differences in outcome could, potentially, result from the different cells used in these studies as well as the heterogenous nature of HLA-DR-alleles expressed by these cells.

We now performed an in-depth analysis of the ligandome of one of the most prevalent HLA-DR molecules in Caucasians, HLA-DRB1*03:01, with associated HLA-DRB3*02:02 molecule derived from homozygous cells (Klitz et al. 2003). The large number of identified ligands (*n* = 16.568) allowed us to accurately determine the frequency of aa residues on the N- and C-terminus. From these 16,568 peptides, 74% were predicted to be derived from HLA-DRB1*03:01. In addition, we analysed the N- and C-terminal residues from ligands eluted from HLA-DRB1*03:01-positive and HLA-DRB1*04:01-positive DCs and compared the conservations to those of a B cell-derived HLA-DRB1*03:01 ligandome. HLA-DRB1*03:01 and HLA-DRB1*04:01 are both implicated in the pathogenesis of various autoimmune diseases in either a predisposing, neutral or protective manner (Ghodke et al. 2005; Matzaraki et al. 2017). The difference in their impact could potentially be traced back to the peptidome they present. Therefore, the knowledge on the characteristics of antigen-processing for each HLA-class II molecule could serve to improve therapies aiming for inducing tolerance towards specific antigens. By knowing how antigens are processed, the amino acid regions surrounding the important T cell epitopes could be optimized for processing. Extending these analyses towards more HLA-DRB1 molecules, but also HLA-DQ molecules, will help pave the way to novel therapies to autoimmune diseases.

Furthermore, there have been several studies that have focused on the residues directly adjacent to the peptide-binding core of the peptide, the peptide-flanking region (PFR) (Ciudad et al. 2017; Holland et al. 2013; Sant’Angelo et al. 2002). These residues have been indicated to play a role in the peptide/HLA-class II-complex stability (Lovitch et al. 2006) and in the recognition of the presented peptides by T cells (Arnold et al. 2002; Holland et al. 2013). It was shown in one of these studies that the PFR was highly conserved among several HLA-class II alleles with the presence of mainly acid residues and proline at the N-terminal PFR and basic residues at the C-terminal PFR (Ciudad et al. 2017; Godkin et al. 2001). To validate previously acquired and published results, we used our acquired datasets to perform similar PFR analyses.

## Methods

### EBV-transformed B cell culture and lysis

HLA-DRB1*03:01-molecules and HLA-DRB3*02:02-molecules were isolated from the DRB1*03:01-DQB1*02:01-DQA1*05:01-HLA-DRB3*02:02 homozygous EBV-transformed B lymphoblastoid cell line DUCAF. Approximately 9 × 10^7^ cells were grown in IMDM supplemented with L-glutamine and 8% FCS. Subsequently, the cells were harvested, washed with PBS and the cell pellet was stored at − 80 °C. The cells were lysed with 50 ml of lysis buffer (50 mM Tris, 150 mM NaCl, 5 mM EDTA, 0.5% Nonidet P-40 and a complete protease inhibitor mix (Roche)). To remove the nuclei and insoluble material, the lysate was centrifuged for 60 min at 10,000*g*. Lysates were stored at − 80 °C.

### Isolation of monocytes from healthy donors and generation of dendritic cells

Isolation and generation of monocyte-derived DC from homozygous HLA-DRB1*03:01 or HLA-DRB1*04:01 healthy donors were performed as described previously (Chicz et al. 1993). Human peripheral blood mononuclear cells (PBMCs) were isolated by Ficoll gradient from three HLA-DRB1-typed buffy coats per HLA-DR haplotype, obtained from healthy blood donors, and subsequently, CD14^+^ monocytes were isolated and cultivated with GM-CSF (800 U/ml) and IL-4 (500 U/ml) (Invitrogen, Breda, The Netherlands) for 6 days to obtain immature DC. The iDC were matured by incubating 0.5 × 10^6^ DC/well in a 24-well plate with LPS (100 ng/ml) and IFN-γ (1000 U/ml) for 24 h. After 30 h, ~ 20 × 10^6 mature (m)DCs were harvested (obtaining approximately 40 × 10^6^), washed three times with and lysed in 1-ml lysis buffer (50 mM Tris, 150 mM NaCl, 5 mM EDTA, 0.5% zwitterion, 10 mM iodoacetamide and a complete protease inhibitor mix (Roche)) and subsequently high-speed centrifuged for 60 min at 10,000×*g* to remove nuclei and insoluble material.

### Peptide elution and isolation from affinity-purified HLA-DR molecules

A total of 2.5 mg pan-HLA-DR antibody (B8.11.2) was coupled to 1-ml protein-A-Sepharose CL4B beads by dimethyl pimelimidate crosslinking (Schneider et al. 1982). Beads were prewashed with lysis buffer by gravitation in small columns. DUCAF and DC lysates were precleared using Sepharose CL4B (GE Healthcare) beads and HLA molecules were isolated using 100 μl of Ab-protA-Sepharose beads for each 100 × 10^6^ cells. After isolation, the beads were washed with lysis buffer followed by washing steps with low salt buffer (120 mM NaCl, 20 mM Tris-HCl, pH 8.0), high salt buffer (1 M NaCl, 20 mM Tris-HCl, pH 8.0), no salt buffer (20 mM Tris-HCl, pH 8.0) and low Tris buffer (10 mM Tris-HCl, pH 8.0). The peptides were subsequently eluted with 10% acetic acid.

### Peptide identification by mass spectrometry

Mass spectrometry (MS) analysis of HLA-eluted peptides was performed as described previously (van Lummel et al. 2011) with some modifications. After elution, HLA molecules and HLA-binding peptides were separated by selective elution from a small C18 column (Oasis, Waters) in two fractions with 20% and 30% acetonitrile (Lecaille et al. 2002). DUCAF peptides were pre-fractionated into 25 fractions using SCX HPLC. Subsequently, the HLA-peptides were analysed via on-line C18-nano-HPLC-MS with a system consisting of an Easy nLC 1000 gradient HPLC system (Thermo, Bremen, Germany) and a Q-Exactive mass spectrometer (Thermo). Fractions were injected onto a homemade precolumn (100 μm × 15 mm; Reprosil-Pur C18-AQ 3 μm, Dr. Maisch, Ammerbuch, Germany) and eluted via a homemade analytical nano-HPLC column (15 cm × 50 μm; Reprosil-Pur C18-AQ 3 um). The gradient was run from 0 to 30% solvent B (10/90/0.1 water/ACN/FA *v*/*v*/*v*) in 120 min. The nano-HPLC column was drawn to a tip of ∼ 5 μm and acted as the electrospray needle of the MS source. The Q-Exactive mass spectrometer was operated in top 10-mode. Parameters were resolution 70,000 at an AGC target value of 3 million maximum fill time of 100 ms (full scan), and resolution 35,000 at an AGC target value of 1 million/maximum fill time of 128 ms for MS/MS at an intensity threshold of 785,000. Apex trigger was set to 1 to 5 s, and allowed charges were 1–3. All fractions were measured twice. In a post-analysis process, raw data were converted to peak lists using Proteome Discoverer 2.1. For peptide identification, MS/MS spectra were submitted to the *Homo sapiens* database (67,211 entries) using Mascot Version 2.2.04 (Matrix Science) with the following settings: 10 ppm and 20 millimass units deviation for precursor and fragment masses, respectively; no enzyme was specified. In percolator, an FDR of 1% was set, with the additional condition of a mascot score of at least 35.

The mass spectrometry proteomics data have been deposited to the ProteomeXchange Consortium via the PRIDE (Perez-Riverol et al. 2019) partner repository with the dataset identifier PXD014253 (Deutsch et al. 2017).

### Gibbs clustering

The peptides were clustered using the Gibbs Clustering tool available online (Gibbs Cluster 1.1 server) (Andreatta and Lund 2013). The number of clusters was set to 1–4 and motif length to 9 amino acids. The remainder of the settings was used as default.

http://www.cbs.dtu.dk/services/GibbsCluster/

### NetMHCIIPan 3.2

The binding cores of the HLA-DR-derived ligandomes were predicted using NetMHCIIPan 3.2 logarithm applying the default settings (Jensen et al. 2018). The resulting binding motif was compared to the binding motif described in the SYFPEITHI database (Rammensee et al. 1999).

http://www.cbs.dtu.dk/services/NetMHCIIpan/

### iceLogo

Sequence logos were generated by plotting the amino acid sequences against a positive reference set of the *Homo sapiens* proteome using iceLogo version 1.3.8 (Colaert et al. 2009).

### Statistical analysis

Statistical analysis was performed with Stata SE 14.1 (StataCorp LLC). To test for significance, the Chi-square test was performed. The Bonferroni method was used to correct for multiple testing for all statistical analyses (5 amino acid groups × 3 pairwise comparisons × 2 termini × 4 HLA-DRB molecules). The *p* value was adjusted according to the 120 tests performed leading to a significant threshold of *p* = 0.00042.

## Results

### Determining the HLA-DR-derived ligandome from B cell line

To establish the HLA-DR ligandome, HLA-DR/peptide complexes were isolated from the EBV-transformed B cell line DUCAF. This resulted in the identification of one of the largest datasets of HLA-class II-derived ligands (16.568 unique ligands with a false discovery rate of 1% and mascot ion score of 35 and higher) described for a two associated HLA-class II molecules. This set of ligands allowed us to perform a detailed characterization of the HLA-class II ligandome.

To quantify the variety of identified ligands, the theoretical isoelectric points (pI) and gravy index, a measure of hydrophobicity was calculated for all identified ligands. These analyses showed that ligands with a broad spectrum of hydrophobicity and pI were identified, indicating that no strong bias was introduced during the sample pre-treatment and subsequent measurements.

### Characteristics of the DUCAF ligandome

**Fig. 1:**
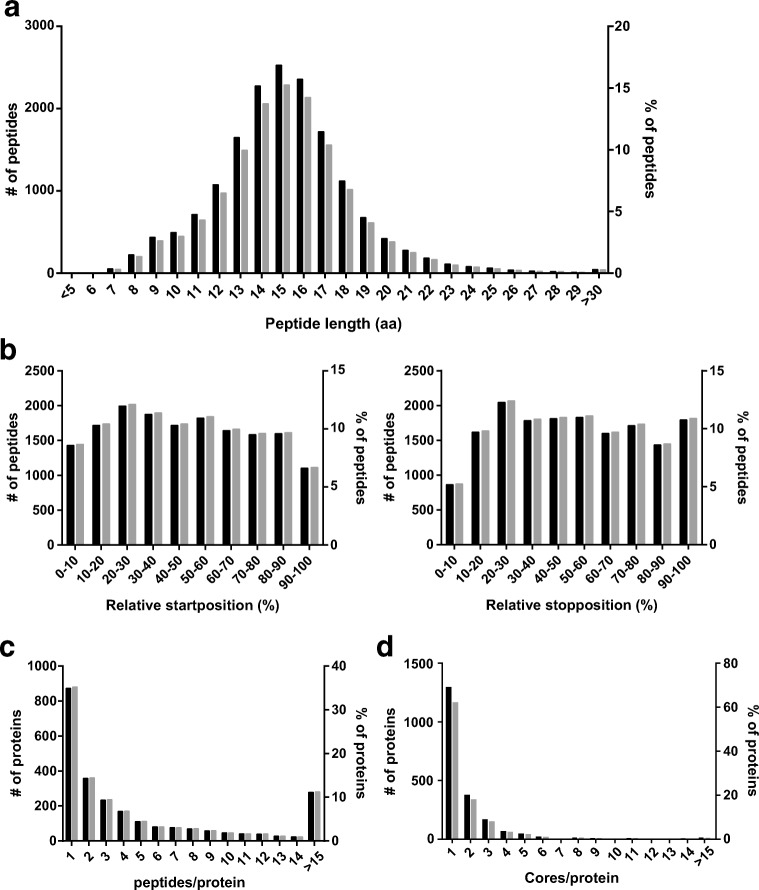
Characteristics of the eluted peptides from HLA-DRB1*03:01^+^ DUCAF cell-line. The peptide length (**a**), position within the protein based on relative start and stop position (**b**), the number of peptides recovered per protein (**c**) and the number of cores recovered per protein (**d**) of the eluted peptidome derived from HLA-DRB1*03:01 is depicted. Both number and percentage of peptides are given, based on 16.568 peptides eluted

Next, the identified ligands were explored in more detail. In Fig. 1a, the length of the ligands is depicted. The peptide lengths found confirmed previous results showing that HLA-class II ligands are heterogeneous in size with 83% of the ligands varying in length between 12 and 20 aa (Chicz et al. 1992; Hunt et al. 1992).

Furthermore, the location of the peptide within the protein was characterised. The position of the peptide within the protein did not show an obvious bias, though peptides derived from the carboxy terminal of the proteins were slightly underrepresented in the ligandome (Fig. 1b). Moreover, the 16.568 ligands identified were derived from 2.475 different proteins. When the number of ligands was plotted against the different proteins, most of the proteins (84%) were represented with one to ten unique peptides (Fig. 1c). As previously described, certain proteins, including different HLA proteins, were clearly overrepresented (Chicz et al. 1992).

### The HLA-DR ligandome of B cells is conserved on the N- and C-terminus

**Fig. 2:**
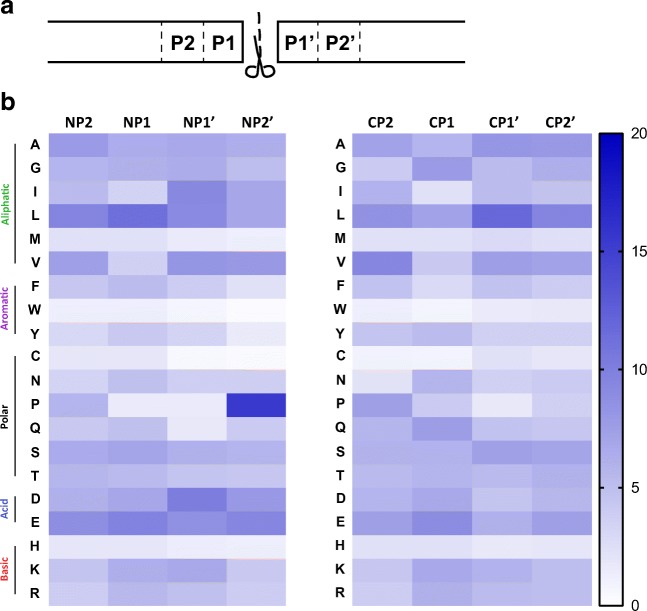
Heatmap of amino acid frequencies at the N- and C-termini of the DUCAF peptidome. A schematic representation of the terminal residues showing the residues analysed (**a**). At each position at the termini (N-terminus: left; C-terminus: right), the frequency in percentages is shown for all amino acids (**b**). Percentage is determined based on the number of included peptides. The amino acids are subdivided into different categories based on characteristics of the side chains

HLA-class II ligands are generated upon lysosome degradation of proteins and are therefore the result of the action of specific proteases and peptidases within the HLA-class II compartment (Chapman 2006). These proteases and peptidases disrupt the bond between two amino acids on two locations (N-terminal and C-terminal), releasing the peptide. To analyse whether the action of these proteases/peptidases would be reflected in conservations of particular aa residues in the N- and C-terminal peptide-positions, the frequency of individual aa residues was determined on eight positions located on the N- and C-termini: the four residues at the N-terminal (designated NP2, NP1, NP1′ and NP2′) and the four C-terminal residues (CP2, CP1, CP1′ and CP2′) of the complete HLA-DR ligandome (Fig. 2a).

To define potential aa conservations, the frequency of each individual aa was determined, as shown in Fig. 2b. Along the N-terminal residues, we observed a relative higher frequency of aliphatic (though not for all) and acidic residues. Proline is highly present at position NP2′, which is in accordance with the data by Ciudad et al. (Fig. 2b). The C-terminal conservations are less evident, but they point towards the conservation of mainly aliphatic and negatively charged residues (Fig. 2b). When these relative frequencies were compared with the frequencies of the residues within the human proteome, again hydrophobic and acidic residues show high frequency compared to the other residues at the N-terminus (supplementary Fig. 1a). Likewise, the differences in frequency are less evident at the C-terminus as the C-terminal residue shows a broader range of residues present (supplementary Fig. 1b).

### N- and C-terminal conservations are similar to HLA-DR3^+^ DCs

As the DUCAF cell line used for the HLA-class II peptide elution might not represent primary cells, the HLA-DR peptidome of monocyte-derived DCs expressing homozygous for HLA-DR3 was also analysed. The peptidome acquired was similar to the DUCAF ligandome. In total, 1287 unique peptides were identified. These peptides display a similar distribution regarding pI and gravy index compared to the ligandome identified from the B cell line (data not shown).

**Fig. 3:**
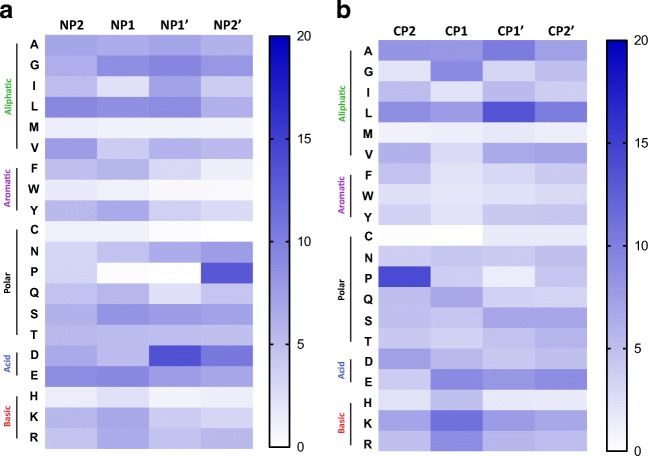
Heatmap of amino acid frequencies at the N- and C-termini of the homozygous HLA-DR3^+^ DC-derived peptidome. At each position at the termini (N-terminus: **a**; C-terminus: **b**), the frequency of each amino acid in percentages is shown for all amino acids (**b**). Percentage is determined based on the number of included peptides. The amino acids are subdivided into different categories based on characteristics of the side chains

To determine the frequencies of the amino acids at the N- and C-termini, the same positions were analysed as was performed for the DUCAF peptidome. When comparing the relative frequencies of the N-terminal residues, similar preferences for aliphatic and acidic residues as well as a disfavour for cysteine, methionine and tryptophan were observed (Fig. 3a). The C-terminus shows an increased frequency in charged residues, both acidic and basic, and aliphatic residues (Fig. 3b). Moreover, proline was highly frequent at the NP2′ and CP2 position, similar to what is found for the DUCAF peptidome and data published by Ciudad et al. (2017)). Together, these observations indicate that the frequencies of aa distribution at the N- and C-termini within the DC ligandome do not differ extensively from the frequencies in the DUCAF ligandome.

### Discriminating HLA-DRB-binding peptide contributions based on predicted binding

The HLA-DR molecules that the DUCAF cell line expresses are besides the HLA-DRB1*03:01, also the HLA-DRB3*02:02 molecule, even though the HLA-DRB3 alleles are expressed in a lower fashion than the HLA-DRB1, depending on cell type (Bontrop et al. 1986; Emery et al. 1993; Stunz et al. 1989). The monoclonal antibody we have used to isolate the HLA-class II molecules from the antigen-presenting cells does not only recognize HLA-DRB1 proteins, but also HLA-DRB3, DRB4 or DRB5 molecules (Bontrop et al. 1990). For the DUCAF cell line, it is described that it co-expresses HLA-DRB3*02:02, a “HLA-DRB > 1” gene expressed on the HLA-DR3-genotype. Therefore, it is likely that the DUCAF dataset includes peptides derived from both HLA-DRB1*03:01 and HLA-DRB3*02:02 (Hurley and Johnson 2001).

In contrast to HLA-class I molecules, HLA-class II molecules have an open configuration allowing the accommodation of a wide variety of peptide lengths (Chicz et al. 1992; Hunt et al. 1992; Stern et al. 1994). The part of the peptide that interacts with the HLA-class II molecules is 9 aa in length (Stern et al. 1994), and HLA-class II-derived ligands usually present in nested sets of ligands of different length with the same 9 aa core sequence.

In order to identify and limit the contamination of the HLA-DRB3-derived peptides within the HLA-DRB1-derived peptidome, and vice versa, the DUCAF peptidome was inserted into the NetMHCIIPan 3.2 server for future analyses. Based on the binding algorithm for HLA-DRB1*03:01 and HLA-DRB3*02:02, the core sequences of the peptides present in the peptidome were predicted using the NetMHCIIPan 3.2 algorithm. Based on these predictions, 74% of the sequences were postulated to be derived from HLA-DRB1*03:01 (data not shown). The remaining 26% were appointed to HLA-DRB3*02:02 as these peptide corresponded best to the HLA-DRB3*02:02 core motifs (data not shown). Together, these analyses resulted in the prediction of 4110 unique ligand-core sequences for HLA-DRB1*03:01, respectively, 1141 for HLA-DRB3*02:02. For 63% and 72% of the proteins, only a single ligand-core sequence was retrieved for HLA-DRB1*03:01 (Fig. 1d) and HLA-DRB3*02:02 (data not shown), respectively.

### Peptide flanking regions of the predicted HLA-DRB1*03:01 peptidome

**Fig. 4:**
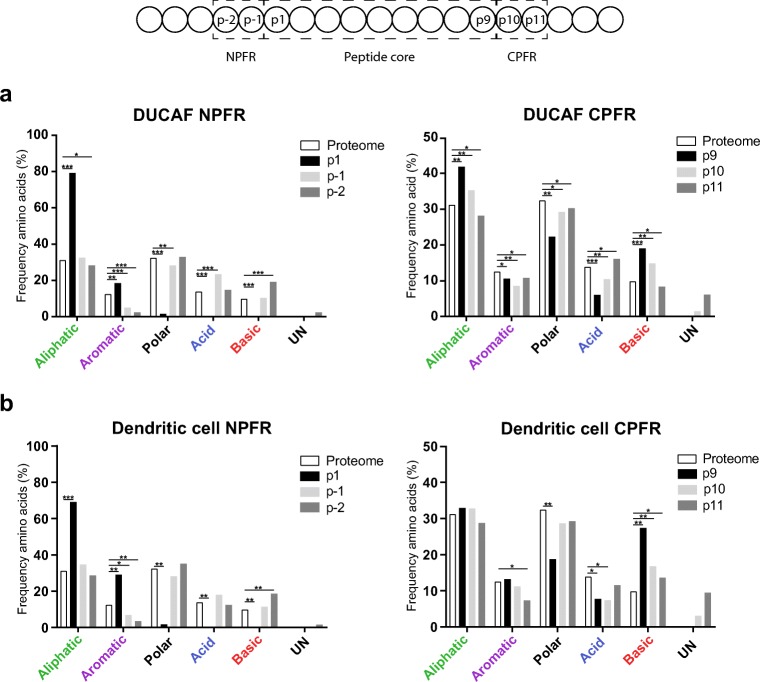
Peptide flanking regions of the HLA-DRB1*03:01 peptidome. The frequencies of the amino acids within the peptide flanking regions of the peptidome derived from either the HLA-DR3 positive B cell line (**a**) or the dendritic cells (**b**) are plotted. The residues flanking the peptide core are represented in different amino acid categories based on characteristics of the side-chains. For both the N-terminal PFR and the C-terminal PFR, three positions are shown of which one is included within the peptide core. Frequencies are compared to the amino acid class frequency within the *Homo sapiens* proteome (white bar). Statistical significance was determined by means of Chi-square tests and subsequent Bonferroni corrections. *P < 0.00042; **P < 1 × 10^10^; ***P < 1 × 10^100^

As mentioned above, PFRs have been described to be involved in both peptide binding to HLA and subsequent recognition by T cells (Arnold et al. 2002; Carson et al. 1997). It has been reported that PFRs are highly similar between multiple HLA-class II molecules (Godkin et al. 2001) as primarily basic residues were found adjacent to the peptide binding core. To further extend these observations, the PFRs were established for the predicted HLA-DRB1*03:01 peptidome. Peptides consisting of 15 residues or more were included in the analysis allowing analyses of all four positions within the PFR as adapted from the aforementioned study (Godkin et al. 2001). As shown in Fig. 4a, preferences for specific residues could not be observed, though the frequency of aromatic and charged residues seem to be reduced and enhanced respectively at the N- and C-terminal PFRs as compared to the frequency in the human proteome. As expected, a similar sequence plot was obtained for the PFRs of the predicted HLA-DRB1*03:01 ligandome from the DCs, reflecting relatively small differences in aa frequencies in the PFRs (Fig. 4b).

### The PFRs of the HLA-DRB3 molecules are highly homologous to the HLA-DRB1 PFRs

**Fig. 5:**
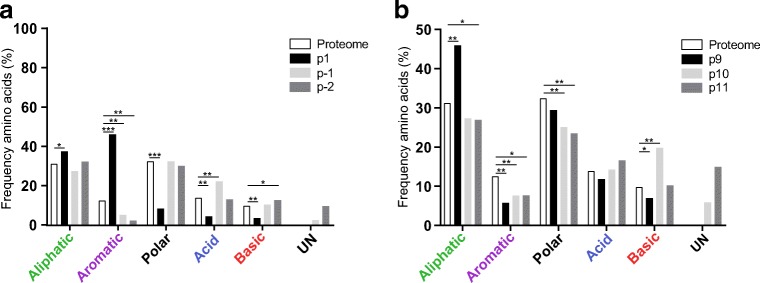
Peptide flanking regions of the HLA-DRB3*02:02 derived peptidome. Peptide cores were determined with NetMHCIIPan 3.2. The frequencies of the amino acids within the PFRs are plotted for the N-terminal PFR (**a**) and the C-terminal PFR (**b**). The residues flanking the peptide core are represented in different amino acid categories based on characteristics of the side-chains. For both the N-terminal PFR and the C-terminal PFR, 3 positions are shown of which one is included within the peptide core (p1 and p9, respectively). Frequencies are compared to the amino acid class frequency within the *Homo sapiens* proteome (white bar). Statistical significance was determined by means of Chi-square tests and subsequent Bonferroni corrections. *P < 0.00042; **P > 1 × 10^10^; ***P > 1 × 10^100^

As shown above, the PFRs for the HLA-DRB1*03:01 molecule derived from two different cell types look identical. Next, a similar analysis was performed for the predicted HLA-DRB3*02:02-ligandome. This analysis, based on the predicted HLA-DRB3*02:02 core sequences derived from the DUCAF cell line, revealed that also in this case, the frequency of aromatic and charged residues is reduced and enhanced, respectively (Fig. 5a and b). Thus, as is shown in Fig. 5, the N-terminal and C-terminal PFR of HLA-DRB3*02:02 only depict minor differences as compared to the PFRs of HLA-DRB1*03:01 for both cell types. Together, these results indicate that the PFRs are not only conserved between cell types, but also between HLA molecules.

The HLA-DR3^+^ DCs most likely also express a secondary HLA-DR molecule. Unfortunately, we do not obtain the information which molecule this is. However, as the PBMCs were derived from a Dutch donor, and the majority of Caucasians with HLA-DRB1*03:01 also express HLA-DRB3*01:01 (Gragert et al. 2013), we have reanalysed the dendritic cell dataset with the HLA-DRB3*01:01 algorithm as described above. Again, the PFR analysis showed corresponding flanking residues on both N- and C-terminal regions (data not shown). Together, these data indicate that the PFRs are conserved across different HLA-DRB alleles.

### Cleavage sites of HLA-DRB1*04/DRB4 peptidomes are comparable to the HLA-DRB1*03:01/DRB3 peptidomes

**Fig. 6:**
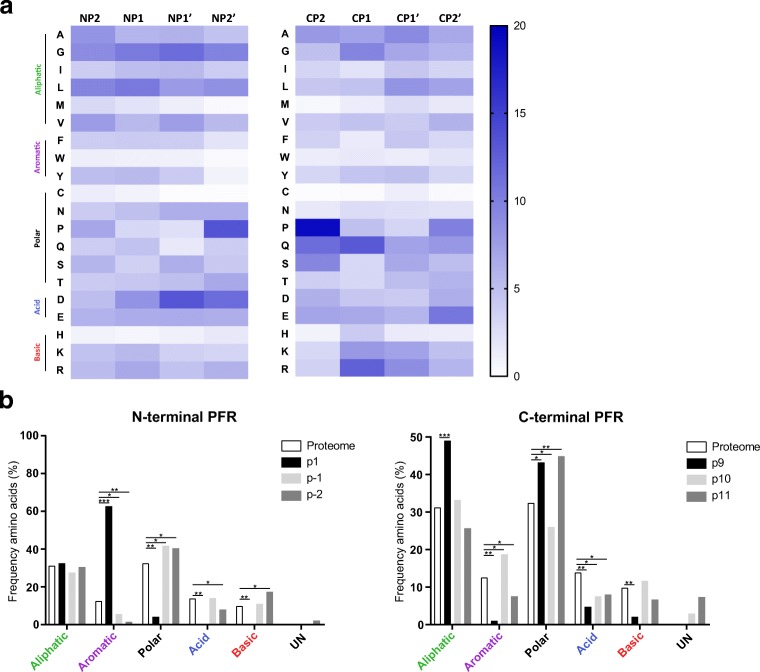
N- and C-terminal residue frequencies and PFR of the HLA-DR4 peptidome. The prevalence of amino acids at the N- and C-termini of peptides eluted from homozygous HLA-DR4-positive dendritic cells is shown as percentage of included peptides in a heatmap (**a**). The amino acids are divided into different categories based on characteristics of the side chains. The frequencies of the amino acids within the peptide flanking regions of the peptide cores (**b**). The residues flanking the peptide core are represented in different amino acid categories based on characteristics of the side-chains. For both the N-terminal PFR and the C-terminal PFR, three positions are shown of which one is included within the peptide core. Frequencies are compared to the amino acid class frequency within the *Homo sapiens* proteome (white bar). Statistical significance was determined by means of Chi-square tests and subsequent Bonferroni corrections. *P < 0.00042; **P > 1 × 10^10^; ***P > 1 × 10^100^

The results described above were conducted in two different cell types carrying the same HLA-molecules showing comparable frequencies of residues at the N- and C-termini. To extend these results not only to different cell types, but also to other HLA-DRB-types, the HLA-DR/peptide complexes were eluted from homozygous HLA-DR4^+^ DCs and analysed for both the N- and C-terminal cleavage sites and PFR. Even though this ligandome was composed of “only” 939 peptides, we were able to analyse the N- and C-terminal cleavage sites, as performed for the DUCAF and DC peptidome. As depicted in Fig. 6a, a preference for aliphatic and acidic residues was found for the N-terminal cleavage site, comparable to the observations made for the HLA-DR3 peptidome, including the high frequency of a proline at NP2′ and aspartic acid at NP1′. Likewise, for the C-terminus, although less evident, higher frequencies are shown for both aliphatic and charged residues, with a high frequency of proline at the CP2 (Fig. 6a). Thus, overall, the frequencies are largely comparable to the results obtained from the HLA-DR3 peptidome.

Similar as for the DUCAF and HLA-DR3+ DC-derived subsets, the peptides were analysed with NetMHCIIPan 3.2 to estimate the contribution of the HLA-DRB1*04:01 by peptides derived from a secondary HLA class II molecule, and to establish where the PFRs are located. Based on the core sequences, the PFRs were analysed to study whether conservations among multiple HLA-class II molecules would be present. Indeed, when compared to the human proteome, the frequency of aromatic residues is lower, but higher for charged residues in both the N-terminal as well as the C-terminal PFR, similar to what is shown for the HLA-DRB1*03:01 peptidome (Fig. 6b). Nevertheless, there seems to be an enrichment for polar residues as well. Therefore, these observations indicate that the PFRs show conservations between different HLA-class II molecules, although more HLA molecules need to be analysed to further validate this notion.

Similar to the HLA-DR3^+^ cells, also HLA-DR4^+^ cells express a secondary HLA-DRB molecule, i.e. HLA-DRB4. Unfortunately, we did not have information regarding the precise HLA-DRB4 allele expressed by the donor. However, as most HLA-DRB4 alleles, except the rare HLA-DRB4*01:04, display a similar binding motif, the core sequences were determined using the information of the ligandome for HLA-DRB4*01:03 to study the HLA-DRB4 PFRs. Similar to what was found for HLA-DRB3, no significant disparities between the PFRs of HLA-DRB1*04:01 and HLA-DRB4 were detected (data not shown).

## Discussion

The human immune system is an intricate network of cells and tissues. Within this network, antigen-presenting cells present protein-derived peptides to T cells, involved in e.g. activation of other immune cells or killing of transformed cells. The peptides presented by HLA molecules provide more insight into the processes underlying antigen processing. Here, we have analysed in detail the HLA-DRB1*03:01 peptidome from different cell types and show that between cell types and HLA-DR-molecules, the N- and C-terminal protease-mediated cleavage-sites are largely conserved. Likewise, conservations within PFRs are present between different cell types as well as different HLA-DR-molecules. Furthermore, we show that HLA-DRB1*03:01 can present a highly diverse range of peptides of mostly between 12 and 20 aa in length that are derived from a broad range of proteins.

Likewise, we show that the electronegative p6 and p9 pocket and the electropositive p4 pocket provide important constraints on the composition of peptides that can be accommodated by HLA-DRB1*03:01. Previous studies described the peptide binding motif of HLA-DRB1*03:01 using peptide substitutions or small sets of eluted peptides (Chicz et al. 1993; Geluk et al. 1992; Geluk et al. 1994; Malcherek et al. 1993). We now extended these studies and constructed a detailed peptide binding motif with 4896 core sequences that could be used in the future to predict potential DR3:T cell epitopes. The latter is of particular interest for auto-immunity as several auto-immune diseases associate with the presence of HLA-DRB1*03:01 (Matzaraki et al. 2017; Price et al. 1999). This core motif is, although more refined, in accordance with the motif described in the SYFPEITHI database.

The HLA-DR3 peptide dataset contains over 16.000 unique peptides and therefore provides a suitable dataset to study different aspects potentially involved in antigen-processing. We have analysed the N- and C-terminal cleavage sites of the peptides to delineate whether there are restrictions to certain aa at specific location that modulate cleavage. Interestingly, restrictions at multiple positions were found, which are likely resulting from the preferences of the proteases or peptidases involved in the antigen processing for HLA-class II. For example, there is a clear increased frequency of proline at the position NP2′ and CP2. In addition, basic residues are more frequent at the C-terminus than at the N-terminus of presented peptides. These preferences were also observed in the peptidome derived from both HLA-DR3 and -DR4 positive DCs. These data are important as they indicate that the preferences observed are present in cells of different origin (B cells and DCs representing lymphoid- and myeloid cell types) as well as across different haplotypes, and hence point to the notion that these are not cell-type or haplotype-specific. Previously, it has been suggested, based upon a limited set of 1319 peptides analysed, that cleavage motifs depend on peptide location in the protein(Ciudad et al. 2017). We could not observe such bias in the ligandomes analysed (data not shown), indicating that preferences for peptide-generation or protease-involvement are not influenced by peptide-location within a protein. Additionally, in a study performed by Ciudad et al., it is mentioned that protein cleaving was most likely executed by cathepsins (D, E, L and S) and matrix metallopeptidases (matrix metallopeptidase-2) (Ciudad et al. 2017) based upon a similarity between protease-specificity and terminal aa of eluted peptides. When comparing the substrate specificities of these proteases with our acquired protein cleavage data, we did not detect such similarity. A possible explanation could be that the pattern that we found for the peptidome only superficially represents all the cleavage sites, while on a more intricate level, different patterns corresponding to different proteases will become apparent. Likewise, experimental conditions or the presence of specific trimming peptidases could also lead to a disturbed representation of the cleavage sites as they have different constraints (for example, the presence of proline could potentially stop peptidases from trimming, whereas a protease would not be influenced). With our dataset, these possibilities are difficult to investigate.

One of the drawbacks that we have encountered with our study is the limited peptide acquisition from the homologous dendritic cells. Including a low number of peptides within the analysis might overestimate the relative enrichment of the amino acids within either the N- and C-terminal cleavage sites or within the PFRs. And although still limited information is present on the ligandome of primary, in vitro generated DCs, a more extended data-set is described which is obtained from HLA-DR peptides expressed by the human cell line MUTZ-3 (Mommen et al. 2016). The ligandome from MUTZ-3 showed homology to our findings and the findings presented by Ciudad et al. that describe the repertoire of HLA-DR-derived peptides from primary human DCs (Ciudad et al. 2017). These observations are relevant as they further emphasize that the antigen-processing machinery for HLA-class II presentation across human cell types is highly conserved. For example, in all cases, a high preference for proline around cleavage sites was noted, possibly reflecting the notion that proline could potentially be involved in abrogating further terminal trimming, rather than being a target for proteases. Nevertheless, also some differences with the ligandome derived from MUTZ-3 were observed as we could confirm the high preference for Leucine at P1 of the N- and C-terminal cleavage sites using DCs generated from the peripheral blood of healthy donors. The reason for this discrepancy is not known, but could be induced by the limited number of peptides used within the analyses.

PFRs might not only be involved in influencing the susceptibility to proteases involved in MHC-ligand generation, they could potentially execute other functions as well. For example, they could interact with parts of the HLA-class II-molecule outside the peptide-binding cleft (Murthy and Stern 1997) or by interacting with other molecules involved in antigen-presentation such as HLA-DM (Lovitch et al. 2006). Therefore, conservation of PFR across MHC-ligands obtained from different MHC molecules does not necessarily reflect the action of common proteases involved in antigen-processing, they might also result from other biologically relevant processes (Godkin et al. 2001; Holland et al. 2013). In this respect, it is intriguing to note that the PFRs from ligands specifically derived from HLA-DRB1*04:01, HLA-DRB1*03:01, but also HLA-DRB3*02:02, are highly homologous. Importantly, the HLA-backbone regions in HLA-DRB1*04, HLA-DRB1*03 and HLA-DRB3*02:02 are identical in the regions thought to interact with the PFRs, pointing to a possible common PFR-interaction-site in these molecules (Bondinas et al. 2007; Murthy and Stern 1997).

A second limitation of this study is the antibody used for the HLA-DR molecule isolation, as we used the monoclonal antibody B8.11.2. This antibody recognizes not only HLA-DRB1 molecules, but also the “HLA-DRB > 1” molecules (DRB3 and DRB4) (Bontrop et al. 1990). Fortunately, the predicted core motifs of the HLA molecules could be well distinguished (Jensen et al. 2018) and PFR analyses were performed for the peptidome likely derived from the different HLA-molecules. Although different core motifs were observed as expected, the PFRs are highly homologous in their overall composition (mainly being aliphatic and polar). Together, these date strengthen the notion that PFRs are conserved across different HLA-DRB molecules (Holland et al. 2013). Nevertheless, it is important to note and keep in mind, that we have analysed the datasets on the basis of predicted peptide-binding core motifs by means of bioinformatical algorithms. In vitro studies are required when defining the biological importance of specific PFRs and terminal cleavage sites of peptides in the context of HLA-class II presentation.

As HLA-DR3 (both HLA-DRB1*03:01 and HLA-DRB3*02:02) has been shown to be involved in the pathogenesis of different autoimmune disease, gaining more insight into the peptidome presented by HLA-DR3 can extend the knowledge on how the HLA molecules participate in the induction (Berrih-Aknin 2014; Matzaraki et al. 2017). Additionally, in the light of the arising patient-specific therapies and therapies concerning the introduction of peripheral tolerance to known antigens, knowledge on HLA-DR3 can prove favourable as T cell epitopes can be predicted more accurately. In some autoimmune diseases where HLA-DR3 appears to be unfavourable in disease onset, HLA-DR4 has been shown to be protective (e.g. Systemic Lupus Erythematosus), or vice versa. The inclusion of the HLA-DR4 peptidome dataset can therefore in turn provide additive information on how HLA-DR3 or HLA-DR4 are implicated and what the differences are between these two molecules. In conclusion, with the HLA-DR3 ligandomes analysed, we can conclude that there are conservations at the N- and C-termini of the peptides eluted from the molecule, which are most likely the results of proteolytic activity and terminal trimming during processing. With the 16.586 different ligands of the HLA-DRB1*03:01/HLA-DRB3*02:02 B cell ligandome now publicly available, additional analyses can be performed related to several immunological questions regarding the role of HLA-DR3 in allergy, autoimmunity, infection and transplant rejection.

## Electronic supplementary material

Supplementary figure 1 **IceLogo plot of amino acid frequencies at the termini of DUCAF peptidome** Frequencies of amino acids are plotted in iceLogo against amino acid frequency in the human proteome for the N terminus (A) and C terminus (B). For all four positions at the N- or C terminus, the size of the character shows frequency; position above or beneath the middle line represents an increase or decrease in prevalence respectively. (PDF 298 kb)
