# Supplementary figures and images for: Ligandomes obtained from different HLA-class II-molecules are homologous for N- and C-terminal residues outside the peptide-binding cleft
Source: Immunogenetics. 2019 Sep 13;71(8):519–30. doi: 10.1007/s00251-019-01129-6 (PMC6790208; doi:10.1007/s00251-019-01129-6)

**A**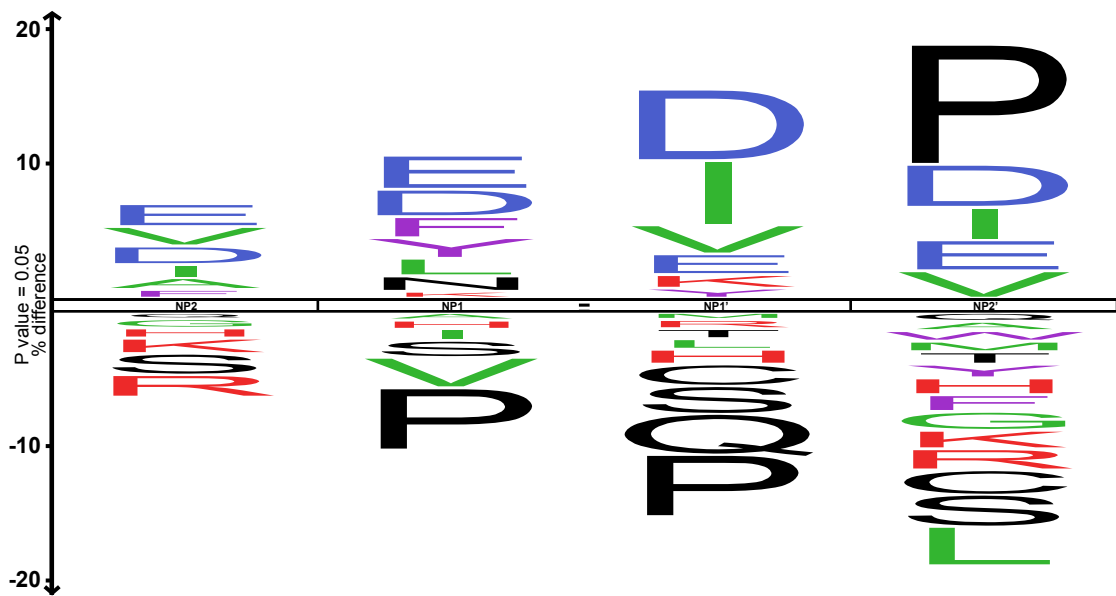**B**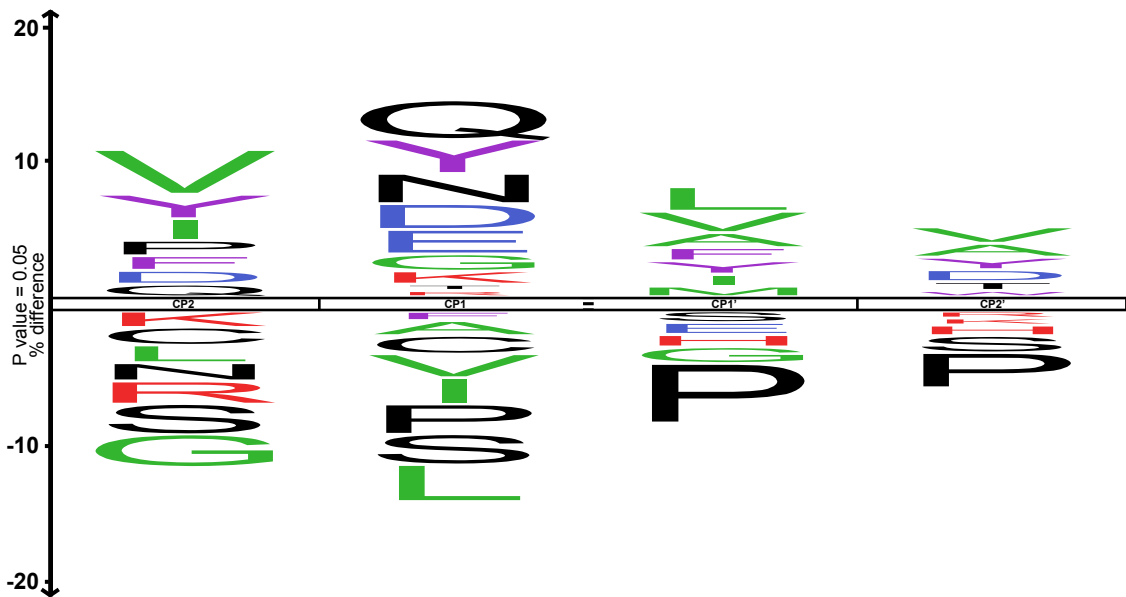

Supplement: Supplementary file 1 — IceLogo plot of amino acid frequencies at the termini of DUCAF peptidome Frequencies of amino acids are plotted in iceLogo against amino acid frequency in the human proteome for the N terminus (A) and C terminus (B). For all four positions at the N- or C terminus, the size of the character shows frequency; position above or beneath the middle line represents an increase or decrease in prevalence respectively. (PDF 298 kb) [file 251_2019_1129_MOESM1_ESM.pdf]
